# A Novel Sample Selection Strategy for Imbalanced Data of Biomedical Event Extraction with Joint Scoring Mechanism

**DOI:** 10.1155/2016/7536494

**Published:** 2016-12-14

**Authors:** Yang Lu, Xiaolei Ma, Yinan Lu, Yuxin Zhou, Zhili Pei

**Affiliations:** ^1^College of Computer Science and Technology, Jilin University, Changchun, Jilin 130000, China; ^2^Library, Inner Mongolia University for Nationalities, Tongliao, Inner Mongolia 028000, China; ^3^College of Computer Science and Technology, Inner Mongolia University for Nationalities, Tongliao, Inner Mongolia 028000, China

## Abstract

Biomedical event extraction is an important and difficult task in bioinformatics. With the rapid growth of biomedical literature, the extraction of complex events from unstructured text has attracted more attention. However, the annotated biomedical corpus is highly imbalanced, which affects the performance of the classification algorithms. In this study, a sample selection algorithm based on sequential pattern is proposed to filter negative samples in the training phase. Considering the joint information between the trigger and argument of multiargument events, we extract triplets of multiargument events directly using a support vector machine classifier. A joint scoring mechanism, which is based on sentence similarity and importance of trigger in the training data, is used to correct the predicted results. Experimental results indicate that the proposed method can extract events efficiently.

## 1. Introduction

With the rapid growth of the amount of unstructured or semistructured biomedical literature, researchers need considerable time and effort to read and obtain relevant scientific knowledge. Event extraction from biomedical text is the task of extracting the semantic and role information of biological events, which are often complex structures, such as the relationship between the disease and the drug [1], the relationship between the disease and the gene [2], the interaction between drugs [3], and the interaction between proteins [4, 5]. Automatic extraction of biomedical events can be applied to many biomedical applications. Therefore, biomedical text mining technology is useful for people to find biological information more accurately and effectively.

The official BioNLP challenges have been held for several years since 2009 [6–8]. The BioNLP shared task (BioNLP-ST) [9] aims to extract fine-grained biomolecular events. It includes a number of subtasks, such as GENIA Event Extraction (GE), Cancer Genetics (CG), Pathway Curation (PC), and Gene Regulation Ontology (GRO). Increasing attention has been given to the task of event extraction, where the major task is GE in BioNLP-ST, and it aims to extract structured events from biomedical text such as event types, triggers, and parameters. An event is defined by GE using a formula including an event trigger and one or several arguments. Nine types of events were defined in BioNLP-ST GENIA Event Extraction 2011 (GE'11) and extended to fourteen types of events in BioNLP-ST GENIA Event Extraction 2013 (GE'13). Due to scarce samples of newly defined event types for good training, the study presented in this paper is still based on the nine types defined in GE'11.

**Figure 1:**
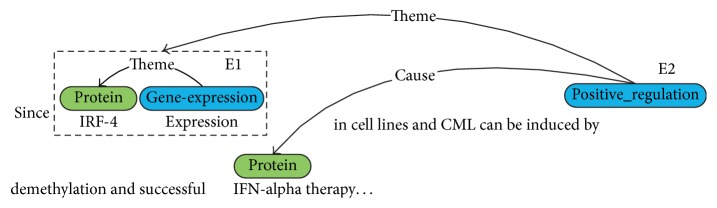
Structured representation of biomedical event.

Table 1 shows the event types, which can be divided into three categories: the simple event class (SVT), Binding event class (BIND), and regulation event class (REG), where there are five simple events, including Gene_expression, Transcription, Protein_catabolism, Localization, and Phosphorylation. Each event has only one argument, that is to say, one theme. Themes in the Binding event comprise up to two arguments. The REG event class includes Regulation, Positive_regulation, and Negative_regulation. They are complex because they have two arguments: a theme and an optional cause.  Figure 1 shows an example of an event where “*IRF-4*” and “*IFN-alpha*” are proteins, “*expression*” and “*induced*” are triggers, and two events can be expressed as {E1: Gene_expression: “*expression*”, Theme: “*IRF-4*”} and {E2: Positive_regulation: “*induced*”, Theme: E1, Cause: “*IFN-alpha*”}. We aim to extract these event structures from the text automatically.

Pattern-based methods are used in biomedical relation extraction [10, 11] but are less used in biomedical event extraction. These methods mainly extract the relations between entities by manually defined patterns and automatically learned patterns from the training data set. Rule-based methods [12–15] and machine learning-based methods [16–18] are the main methods in an event extraction task. Rule-based methods are similar to the pattern-based methods, which manually define syntax rules and learn new rules from the training data. Machine learning-based methods regard the extraction task as a classification problem. The problem of highly unbalanced training data sets in biomedical event extraction is seldom addressed by most systems. The solutions with support vector machines (SVMs) usually use the simple class weighting strategy [19–21]. Other approaches, such as active learning [22, 23] and semisupervised learning [24, 25], solve this problem by increasing the positive sample size. In this study, a sample selection method based on a sequential pattern is proposed to solve the problem of imbalanced data in classification, and a joint scoring mechanism based on sentence semantic similarity and importance of triggers is introduced to correct further false positive predictions.

The paper is organized as follows: related work is presented in Section 2. Our work, the sequence pattern-based sample selection algorithm, detection of multiargument events, and the joint scoring mechanism are presented in Section 3.  Section 4 describes the experiment results in GE'11 and in GE'13 test sets. Finally, a conclusion is presented in Section 5.

## 2. Related Work

Since the organizers of the BioNLP-ST held the first competition on the fine-grained information extraction task of biomedical events in 2009, a variety of methods have been proposed to solve the task. At present, the event extraction systems are mainly divided into two types: rule-based event extraction systems and machine learning-based event extraction systems. The overview papers of BioNLP-ST 2011 and 2013 [7, 8] show that the results of machine learning-based methods are better than the results of rule-based methods.

Rule-based event extraction systems [26–29] are based on sentence structure, grammatical relation, and semantic relation, which make it more flexible. However, the results obtained by those methods have high precision and low recall, which are noticeable in simple event extraction. To improve recall, rule-based event extraction systems are forced to relax constraints in the automatic access of learning rules.

The system based on machine learning is generally divided into three groups. The first group is the pipeline model [30–32], which has an event extraction process that can be divided into three steps. The first step predicts the trigger. The second step is the edge detection and assignment of arguments based on the first step. The final step is the event element detection. The pipeline model in the event extraction task has achieved excellent results, such as the champion of GE'09 [30] (Turku) and the champion of GE'13 [32] (EVEX). Zhou et al. [33] proposed a novel method based on the pipeline model for event trigger identification. They embed the knowledge learned from a large text corpus into word features using neural language modeling. Experimental results show that the *F*-score of event trigger identification improves by 2.5% compared with the approach proposed in [34]. Campos et al. [35] optimized the feature set and training arguments for each event type but only predicted the events in the GE'09 test sets. A linear SVM with “one-versus-the-rest” multiclass strategy is used to solve multiclass and multilabel classification problems based on an imbalanced data set at each stage. Although the performance of the pipeline model is excellent, its time complexity is high and each step is carried out based on the last step, which make its performance dependent on the first step of trigger detection. Thus, if an error occurs at the first step, it will propagate to the next step, thus causing a cascade of errors.

The second group is called the joint model [16, 17], which overcomes the problem mentioned previously. McClosky et al. [36] used the dual-decomposition method for detecting triggers and arguments and extracted the events using the dependence analysis method. Li et al. [37] integrated rich features and word embedding based on the dual-decomposition to extract the biomedical event. However, the optimal state of this joint model requires considering the combination of each token, including the unlikely token in the search space, making its calculation too complicated.

The third group is called the pairwise model [38, 39], which is a combination of the pipeline and joint models that directly extracts trigger and argument instead of detecting the trigger and edge. Considering the relevance of the triggers and arguments, the accuracy of the pairwise model is higher than that of the pipeline model, and it is faster than the joint model in execution time because of the application of a small amount of inference. However, the pairwise model still uses SVM with the “one-versus-the-rest” multiclass strategy to solve multiclass and multilabel classification problems without dealing with the problem of data imbalance.

## 3. Methods

**Figure 2:**
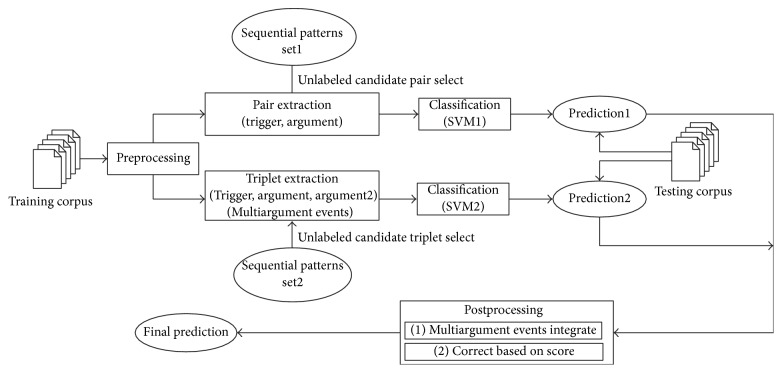
The framework of the proposed method.

This section presents the major steps in the proposed system. The system is based on the pairwise structure in the pairwise model. The event extraction process is summarized in Figure 2. First, the sequential patterns are generated from the training data after text preprocessing. The unlabeled sample pairs in the generation of candidate pair (trigger, argument) will be selected based on the sequence pattern. Then, they will be trained together with the labeled samples. Second, the triplets are extracted directly for multiargument events, and then the predicted results between multiargument and single argument events will be integrated. Finally, the joint scoring mechanism is applied in the postprocessing, and the predicted results are optimized.

### 3.1. Text Preprocessing

Text preprocessing is the first step in natural language processing (NLP). In the preprocessing stage, nonstandard symbols will be removed by NLP tools. We use nltk (nltk.org) to split the words and sentences and use the Charniak–Johnson parser with McClosky's biomedical parsing model (McClosky et al. [36]) to analyze the dependency path. After the sentences and words are split and the full dependence path is obtained, we use the four features' set of the TEES [30] system:

Token features: base stem, character *n*-grams (*n* = {1,2, 3}), POS-tag, and spelling features
Sentence features: the number of candidate entities, bag-of-words
Sentence dependency features: dependency chains features, the shortest dependency path features
External resource features: Wordnet hypernyms

### 3.2. Sample Selection Based on Sequential Pattern

Sequential pattern mining is one of the most important research subjects in the field of data mining. It aims to find frequent subsequences or sequential events that satisfy the minimum support. There are many efficient sequential pattern mining algorithms that are widely used.

Given a sequence database *S*, which is a set of different sequences, let *S* = {*s*_1_, *s*_2_,…, *s*_*n*_}, where each sequence *s*_*x*_ is an ordered list of items and *s*_*x*_ = {*x*_1_, *x*_2_,…, *x*_*p*_}, where *x*_*i*_ is an item and *p* is the number of items. The length of *s*_*x*_ sequence is *p*. Let sequences *s*_1_ = {*a*_1_, *a*_2_,…, *a*_*i*_} and s_2_ = {b_1_, b_2_,…, b_j_} be the two sequences in *S*, where *a*_*i*_ and *b*_*j*_ are items. If there exist some integers 1 ≤ *m*_1_ < *m*_2_ < ⋯<*m*_*n*_ ≤ *j*, make *a*_1_⊆*b*_*m*_1__, *a*_2_⊆*b*_*m*_2__,…, *a*_*n*_⊆*b*_*m*_*n*__; then sequence *s*_1_ is called a subsequence of *s*_2_, or *s*_2_ contains *s*_1_, which is denoted as *s*_1_⊆*s*_2_. The support of the sequence *s*_1_ is the number of sequences in the sequence database* S* containing s_1_, denoted as *Support*(*s*_1_). Given a minimum support threshold minsup, if the support *s*_1_ is no less than *minsup* in *S*, sequence *s*_1_ is called a frequent sequential pattern in *S*, which is denoted as *F*_*s*_1__, *F*_*s*_1__ = {*s*_1_∣*Support*(*s*_1_) ≥ *minsup*, *s*_1_⊆*s*_2_, *s*_2_ ∈ *S*}. In this study, the sequence patterns are generated to select samples in combination with the* PrefixSpan* algorithm [40]. The* PrefixSpan* algorithm uses the principle “divide and conquer” by generating a prefix pattern and then connects it with the suffix pattern to obtain the sequential patterns, thus avoiding generating candidate sequences.

#### 3.2.1. Extraction of Sequential Patterns in Texts

A sequence database *DS* is constructed. We denote *C*_*S*_ = {*c*_*i*_}, *i* = 1,2,…, *n*, as the set of candidate triggers which come from the trigger dictionary and *A*_*S*_ = {*a*_*j*_}, *j* = 1,2,…, *m*, as the set of candidate arguments which come from the training corpus. The set of pair (trigger, argument) is denoted as *P*_*S*_ = {(*c*_*i*_, *a*_*j*_)∣(*c*_*i*_, *a*_*j*_) ∈ *C*_*S*_ × *A*_*S*_, *c*_*i*_ ≠ *a*_*j*_}. The dependency path between the labeled candidate pairs (*c*_*i*_, *a*_*j*_) from the training data is extracted, and it consists of typed dependency sequence. For example, the sequence *s*_1_ = {*nsubj*, *prep*_*by*, *nn*} is the dependency path between the labeled candidate pair (*c*_*i*_, *a*_*j*_) (the dependency path refers to typed dependency sequence from *c*_*i*_ to *a*_*j*_). The dependency paths from all labeled candidate pairs make up the sequence database *DS*, where *s*_1_ is one of the sequences.  Table 2 shows part of the sequences in *DS* and frequent subsequences. The sequence *s*_3_ is shown as a subsequence of *s*_1_ and *s*_5_; therefore, *Support*(*s*_3_) is 3 in* DS*. If we set *minsup* = 3, we obtain* s*_*3*_ as a frequent sequential pattern in* DS*.

We select each unlabeled candidate pair (c_i_, a_j_) based on the frequent sequential patterns. The output frequent patterns set is denoted as *LS*, and the typed dependency sequence of the pair (*c*_*i*_, *a*_*j*_) is denoted as *L*_(*c*_*i*_,*a*_*j*_)_. If *L*_(*c*_*i*_,*a*_*j*_)_ contains enough number of sequences in *LS*, where the number is denoted as *F*_(*c*_*i*_,*a*_*j*_)_, Θ is a threshold; if *F*_(*c*_*i*_,*a*_*j*_)_ > Θ, then the pair is selected. This makes selecting a threshold for selecting the unlabeled candidate pair. We select the suitable threshold with respect to the performance on the development set and discuss threshold in more detail in the experiment section (Section 4.1.1). The formula is as follows:

(1) $$\begin{array}{c} F_{\left(c_{i},a_{j}\right)}=\sum s_{i},\;s_{i}\subseteq L_{\left(c_{i},a_{j}\right)},\;\;s_{i}\in LS. \end{array}$$

For example, let sequences *α* = {*prep*_*of*, *nn*}, *β* = {*nn*}, and *γ* = {*nsubj*, *prep*_*of*, *nn*} be the three frequent sequences in* LS*, and sequence *γ* is the typed dependency sequence of the candidate pair (*c*_*i*_, *a*_*j*_). The sequences *α* and *β* are the subsequences of *γ*. Set* threshold* as 2 and obtain *F*_(*c*_*i*_,*a*_*j*_)_ > 2, where the candidate pair (*c*_*i*_, *a*_*j*_) is selected.  Algorithm 1 summarizes sample selection based on sequential pattern algorithm.

### 3.3. Detection of Multiargument Events

BIND and REG event classes are more complex because of the involvement of primary and secondary arguments. In the primary arguments, some are unary and others can be related to two arguments. In this study, only the primary arguments (theme (protein/event), cause (protein/event), and theme (protein) +) are taken into account. To better solve the multiargument events, which can be represented as a triplet (trigger, argument, argument2), we propose a method that extracts triplet relations directly.

For the single argument events, the pairs (trigger, argument) are extracted directly. For multiargument events, they are usually detected based on single argument events extraction. Then, the second arguments are assigned and reclassified to predict. This approach will result in cascading errors. Considering the Binding multiargument event of the pairwise model [32] as an example, the detect process mainly included two phases: (1) detected pairs. For example, there are two pairs (*c*_*i*_, *a*_*j*_) and (*c*_*i*_, *a*_*k*_) that are extracted from the same sentence with the same trigger *c*_*i*_ and labeled as Binding type. (2) Based on the previous step, evaluate the potential triplet using a dedicated classifier. For example, the triplet (*c*_*i*_, *a*_*j*_, *a*_*k*_) is evaluated as a potential Binding event. Here, *c*_*i*_ is a trigger labeled previously; *a*_*j*_ and *a*_*k*_ are proteins labeled previously in pairs. The result of the first step affects the second step. If pair (*c*_*i*_, *a*_*j*_) or pair (*c*_*i*_, *a*_*k*_) is not labeled, triplet (*c*_*i*_, *a*_*j*_, *a*_*k*_) will not be detected too. Therefore, for the events that include two arguments, the solution is to extract triplet relations directly. This method uses a single dictionary and classifier for multiargument events. The detail is as follows:

(1) Generate dictionary for BIND event class and REG event class from the training data.
(2) Select candidate triplets based on the sequential pattern.
(3) Train the model with an SVM classifier.
(4) Predict the set of triplets {(*c*_*i*_, *a*_*j*_, *a*_*k*_)∣*j* ≠ *k*, *c*_*i*_ ∈ *C*_*S*_, *a*_*i*_ ∈ *A*_*S*_, *a*_*k*_ ∈ *A*_*S*_} after training the model with SVM classifier.

Here, *C*_*S*_ = {*c*_*i*_} is the set of candidate entities and *A*_*S*_ = {*a*_*j*_} is the set of candidate arguments in a sentence *S*, where *a*_*j*_ is labeled proteins and candidate entities from the training data.

For the Binding event, if the triplet (*c*_*i*_, *a*_*j*_, *a*_*k*_) is predicted as true, the single argument of events *e*_*ij*_ = (*Binding*, (*c*_*i*_, *a*_*j*_)) and *e*_*ik*_ = (*Binding*, (*c*_*i*_, *a*_*k*_)) predicted will be removed in the step of integrating the prediction results of the single argument and multiargument of a Binding event. *a*_*k*_ will be output as the cause argument for the REG event class.

If the triplet (*c*_*i*_, *a*_*j*_, *a*_*k*_) is irrelevant to the pair (*c*_*i*_, *a*_*m*_) from the same sentence, it is output directly. Compared to pairwise model, this approach considers joint information among the triplet (trigger, argument, argument2) from the start. It performs better in the multiargument events extraction.

### 3.4. Joint Scoring Mechanism

Due to the introduction of the sequential pattern method to balance the training data, the recall performance is significantly improved. Meanwhile, to correct the false positive examples, a joint scoring mechanism is proposed for the predicted results. The scoring mechanism considers two aspects of sentences: similarity and the importance of trigger, where those less than the threshold will be false positive examples.

Sentence similarity is widely used in the field of online search and question answering system. It is an important research subject in the field of NLP. Here, we use the tool sentence2vec based on convolutional deep structured semantic models (C-DSSM) [41, 42] to calculate the semantic relevance score.

Latent semantic analysis (LSA) is a better-known method for index and retrieval. There are many new methods extending from LSA and C-DSSM is one of them. It combines deep learning with LSA and extends. C-DSSM is mainly used in web search, where it maps the query and the documents to a common semantic space through a nonlinear projection. This model uses a typical convolutional neural network (CNN) architecture to rank the relevant documents. The C-DSSM model is mainly divided into two stages.

**Figure 3:**
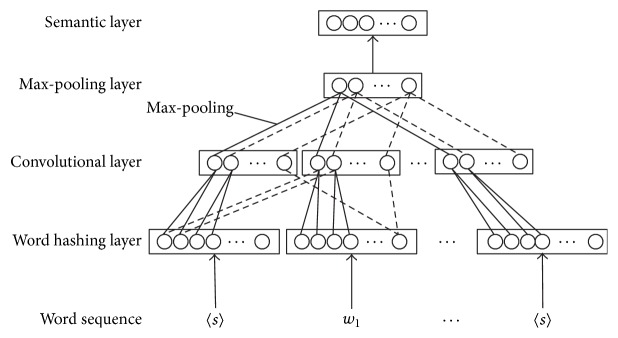
The architecture of the C-DSSM.

(1) Map the word vectors to their corresponding semantic concept vectors. Here, there are three hidden layers in the architecture of CNN. The first layer is word hashing, and it is mainly based on the method of letter *n*-gram. The word hashing method reduces the dimension of the bag-of-words term vectors. After the word hashing layer, it has a convolutional layer that extracts local contextual features. Furthermore, it uses max-pooling technology to integrate local feature vectors into global feature vectors. A high-level semantic feature vector is received at the final semantic layer. The learning of CNN has been effectively improved.  Figure 3 describes the architecture of the C-DSSM.

*x* is denoted as the input term vector, and y is the output vector, *l*_*i*_, *i* = 1,…, *N* − 1, are the intermediate hidden layers, *W*_*i*_ is the *i*th weight matrix, and *b*_*i*_ is the *i*th bias term. Therefore, the problem becomes

(2) l1=W1x,li=fWili−1+bi,i=2,…,N−1,y=fWNlN−1+bN.

(2) Calculate the relevance score between the document and query. By calculating the cosine similarity of the semantic concept vector of 〈query, document〉, the score is obtained and is measured as

(3) $$\begin{array}{c} R\left(\;Q,D\;\right)=\mathrm{cosine}\left(\;y_{Q},y_{D}\;\right)=\frac{y_{Q}^{T}y_{D}}{\left\|y_{Q}\right\|\left\|y_{D}\right\|}. \end{array}$$

The process to calculate the joint score for each predicted result (*typ*, (*t*_*i*_, *a*_*j*_, *a*_*k*_)) is described as follows.

**Step 1 .**

Calculate the similarity between the sentence *s*′ where the predicted result is located and all the related sentences in *d*. Denote *d* = {*s*_1_, *s*_2_,…, *s*_*n*_} as the set of sentences that contain the same trigger with *s*′, and obtain the maximum value

(4) $$\begin{array}{c} Sim\left(\;s^{\prime },d\;\right)=\left\{\begin{array}{cc} \underset{1\leq i\leq n}{\max}\;R\left(s^{\prime },s_{i}\right) & s_{i}\in d,\;\;d\neq \emptyset \\ 0 & \text{otherwise.} \end{array}\right. \end{array}$$

**Step 2 .**

Compute the importance of the trigger, *PR* = {(*typ*, (*t*_*i*_, *a*_*j*_, *a*_*k*_)), *typ* ∈ *eventTyp*, *a*_*j*_ ≠ *∅*}.

(5) P1=ftityp∑typ∈eventTypftityp,P2=∑typ∈eventTypftityp∑ti∈Dfti,Pti=w1P1+w2P2w1+w2,

where *P*_1_ and *P*_2_ are the importance of trigger in training data, *f*(*t*_*i*_^*typ*^) refers to the number of trigger *t*_*i*_ in the event type *typ*, *w*_1_ is the number of trigger *t*_*i*_ belonging to *typ* in the predicted result set *PR*, *w*_2_ is the number of trigger *t*_*i*_ in the predicted result set *PR*, and *eventTyp* is the event type described in Table 1.

**Step 3 .**

Combine *P*_*t*_*i*__ and *Sim*(*t*_*i*_, *a*_*j*_, *a*_*k*_) to score the predicted results. The calculation formula is given as follows:

(6) Scoreti,aj,ak=1−σPti+σSims′,dti,aj,ak∈s′,

where *σ* represents a weight. The sentence similarity computation is based on the semantic analysis, which can correct the false positive example very well. Therefore, weight *σ* in formula (6) will be given a higher value.

**Step 4 .**

Given a threshold *δ*, if *Score*(*t*_*i*_, *a*_*j*_, *a*_*k*_) < *δ*, the example is considered as negative.

## 4. Experiments

### 4.1. Experimental Setup

The experiments on GE'11 and GE'13 corpus are conducted. Nine types of events were defined in GE'11 and extended to fourteen types of events in GE'13. The study presented in this paper is still based on the nine types defined in GE'11. The data sets of GE'11 and GE'13 are different. No abstracts were included in GE'13, and the number of papers in GE'13 is more than that of the papers in GE'11.  Table 3 shows the statistics on the different data sets. We merge GE'11 and GE'13 training data and development data as the final training data. The final training data, which eliminate duplicate papers, contain 16375 events. All parameters of our system have been optimized on the development set. The approximate span/approximate recursive assessment is reported using the online tool provided by the shared task organizers. Our method is mainly divided into three steps: sample selection based on the sequential pattern for imbalanced data, pairs and triplets extraction for multiargument events and integration between them, and a joint scoring mechanism based on sentence semantic similarity and trigger importance.

#### 4.1.1. Filter Imbalanced Data

In the selection stage for sequential patterns sample, we optimize the parameters of the sequence pattern on the GE'11 development set, where a different sequence pattern of minimum support and threshold results in different *F*-score. We merge GE'11 and GE'13 training data as training data of optimized parameters. We aim to improve the recall through sequential pattern sample selection in the event extraction and then to improve the precision of each event while maintaining the recall, thus improving the final *F*-score.

**Figure 4:**
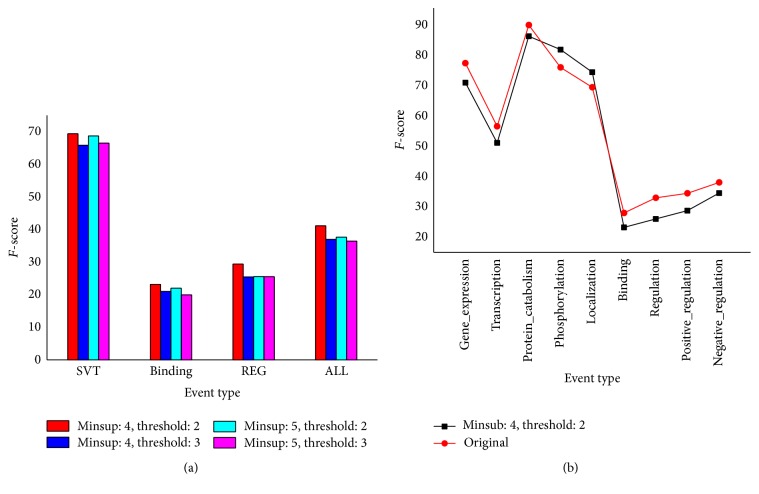
(a) The *F*-score of four sequences on the GE'11 development data. (b) Comparison of the *F*-score of the sequence and original for each event.

Table 4 shows the ratio of the positive and negative samples after different minimum support and threshold selection by the sequential pattern on the parameter training data. Here, we use the number of events as the number of samples. The ratio of positive samples and negative samples is 1 : 13.163 in the annotated corpus. Reducing the negative samples too much or too little will lead to offset data, thus affecting the classifier performance, which is not our original intention. Therefore, we choose to reduce about 50% of the negative samples by setting a minimum support and threshold (*minsup*, Θ) = {(*minsup*, Θ)∣*minsup* ∈ {4,5}, Θ ∈ {2,3}}.  Figure 4(a) shows the *F*-score of the four sequences in the GE'11 development set; when the minimum support is 4 and the threshold is 2, the *F*-score of each event is significantly higher than that of the other sequences.  Table 5 shows the ratio of the positive and negative samples after different minimum support and threshold selection by the sequential pattern on the final training data. From Tables 4 and 5, the ratio of the positive and negative samples is very close. Therefore, we will use the sequence where the minimize support is 4 and the threshold is 2 on GE'11 and GE'13 test set.  Figure 4(b) shows that almost the *F*-score of every event is less than the original model, which is pairwise model on the GE'11 development set, after the sample selection based on the sequential pattern. Given that we reduce the negative samples resulting in high recall and lower prediction, we propose a joint scoring mechanism to improve the prediction performance.

#### 4.1.2. The Integration of Multiargument Events

The results in Table 6 show that the recall and *F*-score have been significantly improved by extracting directly the triplets of the Binding events. The REG event class includes nested events; therefore, the extraction of the multiargument has high complexity. We only study the Binding events of the multiargument events in this paper. This method, which extracts the triplets directly for the multiargument events, will not cause cascading errors. Therefore, it is effective to extract the triplets of the events.

### 4.2. Results and Discussion

#### 4.2.1. Result on BioNLP-ST GENIA 2011

We evaluate the performance of the method and compare it with the results of other systems.  Table 7 shows the results of the method using the official GE'11 online assessment tool. Given that GE'11 corpus contains abstract and full text, we evaluate the performance on whole, abstract, and full text. The results of abstract and the full text, as well as the whole results, are reported to illustrate that the method is outstanding in classifying events.  Table 7 shows that the *F*-score of the full text is higher than the *F*-score of the abstract, which is 81.32 and 71.44 in the simple event, respectively. However, the *F*-score of the abstract is higher than the *F*-score of the full text in BIND event class, which is 54.17 and 45.93, respectively. The *F*-score of abstract is also higher than the *F*-score of the full text in REG event class, which is 41.36 and 41.10, respectively. The total *F*-score of the full text is higher than that of the abstract, which is 54.23 and 53.64, respectively.  Table 7 also illustrates that the method performs well on full text.

Table 8 shows the comparison results of the proposed method with other GE'11 systems. The results for FAUST, UMass, UTurku, MSR-NLP, and STSS models are reproduced from [7, 24]. Our approach in full text obtains the best *F*-score of 54.23. This score is higher than the best extraction system, such as FAUST of GE'11 (1.56 points), the STSS, and UTurku (about 3.5 points). The performance in precision and recall of full text is also better than that of other systems. However, the precision and recall of SVT and REG event classes are slightly lower than FAUST and UMass in the abstract, but they are higher in Binding events. However, the whole *F*-score is slightly lower than that of the FAUST and UMass and higher than the UTurku, STSS, and MSR-NLP. However, the recall has achieved the highest score, which is mainly due to the sequential pattern sample selection of the unbalanced data.

#### 4.2.2. Result on BioNLP-ST GENIA 2013

The pipeline approaches are the best methods performing on the GE'13, where EVEX is the official winner. We train the model on the training set and development set and evaluate it on the test set using the official GE'13 online assessment tool.  Table 9 shows the evaluation results. GE'13 test data does not contain abstracts; therefore, we evaluate the performance on full papers only.  Table 10 shows the comparison results of our method with other GE'13 systems, including TEES 2.1 and EVEX, because they belong to the pipeline model. We add BioSEM system in the table, which is a rule-based system and has achieved the best results in the Binding events. The results for TEES 2.1, EVEX, and BioSEM are reproduced from [8].  Table 10 indicates that our method is significantly higher than the other systems in the recall rate. Our recall rate is 48.65 and TEES 2.1, EVEX, and BioSEM are 46.60, 45.87, and 42.84, respectively. The *F*-score of our system is 52.17, while the *F*-scores of TEES 2.1, EVEX, and BioSEM are 51.00, 51.24, and 50.94, respectively. Although the total *F*-score of our system is better than the systems previously mentioned, it does not achieve the desired effect on Binding events. This may be the result of a not ideal extracting pair in the Binding simple event, resulting in poor integration of the pairs and triplets in Binding events after the triplets extraction. In general, these results clearly demonstrate the effectiveness of the proposed method.

## 5. Conclusions

In this study, a new event extraction system was introduced. Comparing our system with other event extraction systems, we obtained some positive results. First, we proposed a new method of sample selection based on the sequential pattern to balance the data set, which has played an important role in the process of biomedical event extraction. Second, taking into account the relevance of the trigger and argument of multiargument events, the system extracts the pair (trigger, argument) and triplet (trigger, argument, argument2) at the same time. The integration of the pair and triplet improves the performance of multiargument events prediction, which improves the *F*-score as well. Finally, a joint scoring mechanism based on C-DSSM and the importance of the trigger is proposed to correct the predictions. In general, samples selection based on the sequential pattern achieved the desired effectiveness, and it was combined with the joint scoring mechanism to further improve the performance of the system. The performance of this method was evaluated with extensive experiments. Although our method is a supervised learning method, we provide a new idea on constructing a good predictive model because high recall can be used in disease genes. Although numerous efforts are made, the extraction of complex events is still a huge challenge. In the future, we will further optimize the joint scoring mechanism and integrate external resources into biomedical event extraction through semisupervised or unsupervised approach.

## Figures and Tables

**Algorithm 1:**
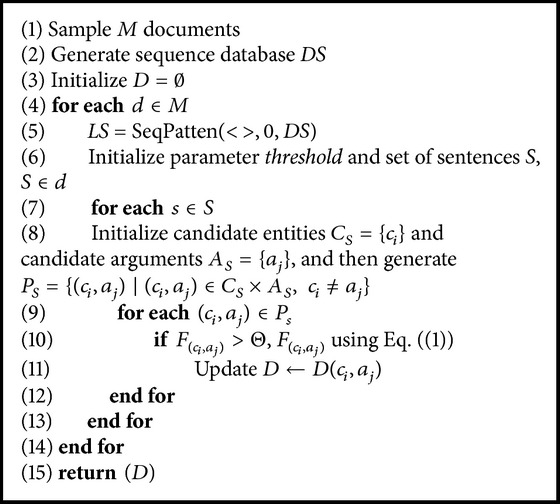
Sample filter.

**Table 1:** Class, event types, and their arguments for the GE task.

Event class	Event type	Primary argument	Secondary argument
SVT	Gene_expression	Theme(P)	
	Transcription	Theme(P)	
	Localization	Theme(P)	AtLoc, ToLoc
	Protein_catabolism	Theme(P)	
	Phosphorylation	Theme(P)	Site

BIND	Binding	Theme(P)+	Site+

REG	Regulation	Theme(P/Ev), Cause(P/Ev)	Site, Csite
	Positive_regulation	Theme(P/Ev), Cause(P/Ev)	Site, Csite
	Negative_regulation	Theme(P/Ev), Cause(P/Ev)	Site, Csite

P is protein; Ev is event.

**Table 2:** Part of frequent sequential patterns.

ID	Sequence database	Frequent sequential pattern
*s*_1_	〈*amod prep_to prep_in nn*〉	〈*amod nn*〉 〈*prep_to nn*〉
*s*_2_	〈*prep_to nn prep_in*〉	
*s*_3_	〈*amod nn*〉	
*s*_4_	〈*prep_to dobj prep_in nn*〉	
*s*_5_	〈*dobj amod prep_to nn*〉	
*s*_6_	〈*prep_to nn*〉	

**Table 3:** Statistics on the GE data sets.

Data set	Papers			Abstracts			Events		
	Training	Devel	Test	Training	Devel	Test	Training	Devel	Test
GE'13	10	10	14	0	0	0	2817	3199	3348
GE'11	5	5	4	800	150	260	10310	4690	5301

Training is training data, Devel is development data, and Test is test data.

**Table 4:** The ratio of the positive and negative samples on the training data.

*X*-*Y*	3-3	4-1	4-2	4-3	5-1	5-2	5-3	6-1	Original
Positive samples	11419								
Negative samples	60742	77829	70901	66809	84910	74851	69180	85247	150308
P : N	1 : 5.319	1 : 6.816	1 : 6.209	1 : 5.851	1 : 7.436	1 : 6.555	1 : 6.058	1 : 7.465	1 : 13.163

*X* is minimum support (*minsup*), *Y* is threshold Θ, and P : N is the ratio of the positive and negative samples.

**Table 5:** The ratio of the positive and negative samples on the final training data.

*X*-*Y*	3-3	4-1	4-2	4-3	5-1	5-2	5-3	6-1	Original
Positive samples	16375								
Negative samples	88054	112961	103045	97192	123122	108571	100341	123528	215383
P : N	1 : 5.377	1 : 6.898	1 : 6.292	1 : 5.935	1 : 7.519	1 : 6.630	1 : 6.128	1 : 7.544	1 : 13.153

*X* is minimum support (*minsup*), *Y* is threshold Θ, and P : N is the ratio of the positive and negative samples.

**Table 6:** Results with/without triplets of the Binding events.

Binding events	Without triplets			With triplets		
	*R*	*P*	*F*	*R*	*P*	*F*
GE'11	42.57	49.76	45.88	47.66	56.52	51.71
GE'13	30.03	31.35	30.67	39.34	44.11	41.59

Performance is shown in recall (*R*), precision (*P*), and *F*-score (*F*).

**Table 7:** Results of the proposed method on GE'11 test set.

Event class	Event type	Whole			Abstract			Full text		
		*R*	*P*	*F*	*R*	*P*	*F*	*R*	*P*	*F*
SVT	*Gene_expression*	75.55	80.19	77.80	72.44	77.71	74.98	83.57	86.35	84.94
	*Transcription*	52.87	68.66	59.74	54.74	70.75	61.73	45.95	60.71	52.31
	*Protein_catabolism*	66.67	76.92	71.43	64.29	81.82	72.00	100.00	50.00	66.67
	*Phosphorylation*	80.00	88.10	83.85	77.04	88.14	82.21	88.00	88.00	88.00
	*Localization*	35.60	86.08	50.37	32.76	95.00	48.72	64.71	57.89	61.11
	Total	68.60	80.34	74.01	64.97	79.34	71.44	79.74	82.97	81.32

BIND	*Binding*	47.66	56.52	51.71	49.57	59.72	54.17	43.06	49.21	45.93

REG	*Regulation*	32.47	47.53	38.58	31.96	51.38	39.41	34.04	39.02	36.36
	*Positive_regulation*	41.03	42.35	41.68	41.10	40.64	40.87	40.87	46.53	43.52
	*Negative_regulation*	38.18	46.38	41.88	40.37	48.57	44.09	33.85	41.94	37.46
	Total	38.97	43.88	41.28	39.32	43.62	41.36	38.20	44.46	41.10

ALL total		50.35	57.79	53.81	49.97	57.90	53.64	51.29	57.52	54.23

Performance is shown in recall (*R*), precision (*P*), and *F*-score (*F*).

**Table 8:** Comparison with other systems on GE'11 test set.

System		Event class			
		SVT	BIND	REG	ALL
FAUST	W	68.47 80.25/73.90	44.20/53.71/48.49	38.02/**54.94**/**44.94**	49.41/**64.75**/**56.04**
	A	**66.16**/**81.04**/**72.85**	45.53/58.09/51.05	39.38/**58.18**/**46.97**	50.00/**67.53**/**57.46**
	F	75.58/78.23/76.88	40.97/44.70/42.75	34.99/**48.24**/40.56	47.92/58.47/52.67

UMass	W	67.01/**81.40**/73.50	42.97/56.42/48.79	37.52/52.67/43.82	48.49/64.08/55.20
	A	64.21/80.74/71.54	43.52/**60.89**/50.76	38.78/55.07/45.51	48.74/65.94/56.05
	F	75.58/**83.14**/79.18	41.67/47.62/44.44	34.72/47.51/40.12	34.72/47.51/40.12

UTurku	W	68.22/76.47/72.11	42.97/43.60/43.28	38.72/47.64/42.72	49.56/57.65/53.30
	A	64.97/76.72/70.36	45.24/50.00/47.50	**40.41**/49.01/44.30	**50.06**/59.48/54.37
	F	78.18/75.82/76.98	37.50/31.76/34.39	34.99/44.46/39.16	48.31/53.38/50.72

MSR-NLP	W	**68.99**/74.30/71.54	42.36/40.47/41.39	36.64/44.08/40.02	48.64/54.71/51.50
	A	65.99/74.71/70.08	43.23/44.51/43.86	37.14/45.38/40.85	48.52/56.47/52.20
	F	78.18/73.24/ 75.63	40.28/32.77/36.14	35.52/41.34/38.21	48.94/50.77/49.84

STSS	W	—	—	—	—
	A	64.97/76.65/70.33	45.24/49.84/47.43	**40.41**/48.83/44.22	50.06/59.33/54.30
	F	78.18/75.63/76.88	37.50/31.58/34.29	34.99/44.69/39.25	48.31/53.43/50.74

Ours	W	68.60/80.34/**74.01**	**47.66**/**56.52**/**51.71**	**38.97**/43.88/41.28	**50.35**/57.79/53.81
	A	64.97/79.34/71.44	**49.57**/59.72/**54.17**	39.32/43.62/41.36	49.97/57.90/53.64
	F	**79.74**/82.97/**81.32**	**43.06**/**49.21**/**45.93**	**38.20**/44.46/**41.10**	**51.29**/57.52/**54.23**

Evaluation results (recall/precision/*F*-score) in whole data set (W), abstracts only (A), and full papers only (F).

**Table 9:** Results of the proposed method on GE'13 test set.

Event class	Event type	*R*	*P*	*F*
SVT	*Gene_expression*	82.88	79.91	81.36
	*Transcription*	52.48	65.43	58.24
	*Protein_catabolism*	64.29	50.00	56.25
	*Phosphorylation*	81.25	76.02	78.55
	*Localization*	31.31	83.78	45.59
	Total	74.12	77.56	75.80

BIND	*Binding*	39.34	44.11	41.59

REG	*Regulation*	23.61	39.08	29.44
	*Positive_regulation*	39.56	46.71	42.84
	*Negative_regulation*	39.73	46.24	42.74
	Total	37.24	45.74	41.05

ALL total		48.65	56.24	52.17

Performance is shown in recall (*R*), precision (*P*), and *F*-score (*F*).

**Table 10:** Comparison with other systems on GE'13 test set.

System	Event class			
	SVT	BIND	REG	ALL
TEES 2.1	**74.52**/77.73/76.09	42.34/44.34/43.32	33.08/44.78/38.05	46.60/56.32/51.00
EVEX	73.82/77.73/75.72	41.14/44.77/42.88	32.41/47.16/38.41	45.87/58.03/51.24
BioSEM	70.09/**85.60**/**77.07**	**47.45**/**52.32**/**49.76**	28.19/**49.06**/35.80	42.84/**62.83**/50.94
Ours	74.12/77.56/75.80	39.34/44.11/41.59	**37.24**/45.74/**41.05**	**48.65**/56.24/**52.17**

Performance is shown in recall (*R*), precision (*P*), and *F*-score (*F*).
